# Needs and challenges among general practitioners in the management of actinic keratosis: a qualitative study

**DOI:** 10.1186/s12875-023-02202-6

**Published:** 2023-12-02

**Authors:** Charlotte Verhoeven, Zilke Claessens, Satish F.K. Lubeek, Henk J. Schers

**Affiliations:** 1 Faculty of Pharmaceutical Sciences, KU Leuven, Leuven, Belgium; 2 Clinical Pharmacology and Pharmacotherapy, Department of Pharmaceutical and Pharmacological Sciences, KU Leuven, Leuven, Belgium; 3 Department of Dermatology, Radboud University Nijmegen Medical Centre, Nijmegen, The Netherlands; 4 Department of Primary and Community Care, Radboud University Nijmegen Medical Centre, 6500 HB Nijmegen, PO Box 9101, The Netherlands

**Keywords:** Actinic keratosis, Primary health care, Healthcare utilization, Disease management, Qualitative research

## Abstract

**Background:**

Because of the increasing incidence of actinic keratosis (AK), optimal use of limited healthcare resources is essential. Although most patients can be managed in primary care, dermatology referrals are common. More profound knowledge of general practitioners’ (GPs) considerations might assist in enhancing AK care.

**Methods:**

The aim of the current study was to gain insight into AK management in primary care by exploring the needs and challenges among GPs in the Netherlands. A qualitative study was conducted based on semi-structured in-depth interviews with 15 conveniently sampled Dutch GPs, focusing on the needs and challenges in AK management. A literature-informed, predefined topic list guided the interviews, which were recorded, transcribed ad verbatim, and thematically analysed using the Framework Method.

**Results:**

All GPs reported AK to be a clinical diagnosis and most GPs indicated that most AK patients could be managed in primary care. Cryotherapy was preferred and experience with 5-FU therapy was limited. Most GPs applied cryotherapy without discussing other treatment options with patients. Reasons for dermatology referrals included an incomplete treatment response, extensive lesions, difficult-to-treat areas, and serious doubts about the diagnosis. GPs reported a need for more education, especially on 5-FU therapy. Their main challenges were dealing with diagnostic uncertainty, treating extensive lesions, managing treatment-related skin reactions, and reconciling patient misconceptions.

**Conclusions:**

This study shows various AK management approaches among Dutch GPs with suboptimal guideline compliance due to diverse underlying barriers. It suggests that more education might contribute to a more standardised and uniform AK management and supports further transition of AK care from hospital to primary care.

**Supplementary Information:**

The online version contains supplementary material available at 10.1186/s12875-023-02202-6.

## Introduction

General practitioners (GPs) are increasingly confronted with patient questions about potentially (pre)malignant skin lesions [1], probably due to an increase in incidence and public awareness of skin cancer [2]. Actinic keratosis (AK) is the most common precancerous skin lesion caused by long-term ultraviolet exposure, and its incidence is also increasing [3, 4]. Prevalence rates of AK derived from population-based studies differ substantially between countries and populations [5–8].

In the Netherlands and the UK, among other countries, both GPs and dermatologists are involved in the management of patients with AK [9]. Dutch and British GPs act as ‘gatekeepers’, ensuring that patients see specialists only for conditions that cannot be managed by a GP and are referred to an appropriate specialist [10]. Although AK is considered a low-risk condition and most lesions can be managed in primary care, Dutch GPs refer 40.000 new patients with AK to a dermatologist annually, accounting for at least 10% of all dermatological visits [11]. In the Netherlands, the total costs for the management of skin tumours, including AK, in secondary care have increased dramatically, from €278 million for 384,390 patients in 2007 to €465 million for 578,355 patients in 2017 [12]. These costs are expected to rise further to €1.35 billion by 2030 [12].

Because of the increasing incidence of AK and related impact on healthcare utilization and costs, optimal use of the limited healthcare resources is essential. Previously, a qualitative study showed a large variation in AK care provided by Dutch GPs, due to a lack of knowledge and experience and low perceived value of AK care [13]. However, this study was conducted before the issuing of the national guideline on ‘suspicious skin abnormalities’ from the Dutch College of General Practitioners (DCGP) in 2017 [14]. It is unclear whether AK care has improved since then. More profound knowledge of GPs’ considerations might assist in enhancing AK care.

Therefore, the aim of the current study was to gain insight into the management of patients with AK after the DCGP guideline implementation by exploring the needs and challenges among Dutch GPs.

## Method

### Study design

A qualitative study was conducted based on semi-structured, oral in-depth interviews with Dutch GPs. Because one-on-one interviews with open-ended questions invite participants to share their views and opinions freely, this approach was considered most appropriate for exploring the needs and challenges among GPs [15]. This study was conducted in accordance with the Consolidated Criteria for Reporting Qualitative Research (COREQ) and the Qualitative Analysis Guide of Leuven (QUAGOL) [15], [16].

### Selection of participants

Participants were selected based on convenience sampling [17]. Approximately 360 GPs subscribed to an online learning platform for dermoscopy in primary care (www.dermatoscopie.nl) were invited to participate in the study. GPs could register for participation by contacting the researchers. The aim was to recruit 9–17 GPs, as most studies show saturation after these numbers of participants [18].

### Data collection, processing and analysis

Video interviews were conducted in March-April 2022 by an independent researcher (CV). Based on an extensive literature review, a topic list related to the following main themes was compiled: general information (e.g. knowledge and experience regarding AK management), diagnosis (e.g. dermoscopy use, reasons for biopsy), treatment (e.g. preferred therapy, experience with different treatment modalities), and follow-up (e.g. appointment scheduling, reasons for referral). This predefined topic list guided the interviews (see *Supplementary Material*).

All interviews were recorded and, subsequently, transcribed ad verbatim by a professional transcriber affiliated with Amberscript (Amberscript Global BV, Amsterdam, The Netherlands). Transcripts were pseudo-anonymised, checked and, if necessary, corrected by one of the researchers (CV). Thereafter, transcripts were uploaded in NVivo (Lumivero, Denver, US) and thematically analysed using the Framework Method [19]. The framework was constructed using both deductive and inductive coding. On the basis of the literature, some predefined themes were selected and molded into a topic list. In the first step, familiarization with the first 5 interviews took place by listening and relistening to the recordings and taking notes of the initial findings. Thereafter, the process of coding started with applying the predefined codes (deductive coding) and looking for new themes (inductive coding). This process led to a coding tree with clustered codes. This coding tree was then applied to the remaining interviews, also leaving room for new open codes. Finally, the data were mapped and interpreted. After 15 interviews, no new information was surfaced, meaning that thematic saturation was reached in all main themes [20].

## Results

Of the 15 participating GPs, 9 were female and the median age was 35 (range: 32–56) years. More than half of the GPs were GP partners, whereas the others were GP locums. GPs. The participants worked in various regions of the country. The median number of years of working experience was 8.5 (range: 1–23) and the estimated number of patients consulting the GP because of AK ranged from 1 to 2 per month to 3–4 per week. All participants were aware of the existence of the DCGP guideline.

## Management

### Diagnosis

All GPs reported diagnosing AK based on its clinical manifestations. To increase diagnostic certainty, nearly half of the GPs stated using a dermatoscope. The majority of GPs stated using teledermatology. Diagnostic uncertainty, lack of treatment effect or atypical localization of the lesion were indicated to be the main reasons for performing a skin biopsy. GPs mentioned that patients usually consult the GP to assess a skin lesion that they are worried about.

‘A biopsy, that I really perform when I think I am in doubt. And yes, I think we often say: we’ll just treat it with nitrogen once if the suspicion is very high. If it doesn’t respond, then you can still perform a biopsy.’ [GP #2].

Only when a suspicious skin lesion is clearly visible, GPs proactively assessed this lesion. If deemed necessary, a new appointment was scheduled for assessment.

### Treatment

All GPs addressed cryotherapy as the preferred treatment. Experience with 5-fluorouracil (5-FU) therapy was limited to a few GPs. GPs not applying 5-FU therapy refrained from it because of seeing few patients with extensive lesions or fearing severe treatment-related skin reactions. Low compliance rates due to the long treatment period and difficulties in clearly explaining the therapy to patients were also reported motives for not treating patients with 5-FU. GPs expressed openness to the use of other treatment modalities, such as daylight photodynamic therapy. When choosing between cryotherapy and 5-FU, GPs addressed the number of lesions as the main consideration. Localization and patient preference were also reported to play an important role. Regarding patients with less than 5 lesions, all GPs stated using cryotherapy and most of them stated not discussing other treatment options with the patient. Furthermore, it was mentioned that the choice of not treating AK was rarely discussed with the patient.

‘Mostly, I use cryotherapy. And particularly because that eh, you can do that right away. It’s simple. Patients also feel right away that something is happening.’ [GP #3].

‘If there are two or three lesions, I say: “We’re just going to treat it with nitrogen.” Then I don’t actually discuss the cream that much. […] And if it’s eh, multiple lesions, in that sense I do involve the patient and I say: “Well, I can also prescribe that, or I can refer you to a dermatologist.”’ [GP #4].

### Follow-up

Most GPs indicated not routinely scheduling a check-up appointment after cryotherapy. Instead, patients were advised to monitor the treated area of the skin closely and to book an appointment if healing failed to occur. If deemed necessary, cryotherapy was repeated. Most GPs who treated patients with 5-FU employed a stricter follow-up regimen in these patients by scheduling a check-up or telephone appointment 2 weeks after treatment. An incomplete treatment response was addressed as the main reason to refer patients to a dermatologist. Other reported motives for referral were extensive lesions, difficult-to-treat areas (e.g. on nose, ear), serious doubts about the diagnosis, and prior consultation of a dermatologist.

‘And I often have the people who apply Efudix [5-FU] come back eh, after two weeks, just to take a quick look of eh, are you keeping up, how is the treatment eh, what does it do to your skin eh, did you expect it that way or not?’ [GP #2].

### Needs and challenges

All GPs advocated greater attention to dermatology education in medical school and GP training curricula. Some of the GPs expressed a need for continuing education, especially on 5-FU. Lack of photographic and dermoscopic images was considered a drawback of the current DCGP guideline. The main challenges in AK management as perceived by GPs were dealing with diagnostic uncertainty, treating extensive lesions, managing treatment-related skin reactions, and reconciling patient misconceptions (e.g. treatment-related skin reactions, request for direct referral to a dermatologist without knowing what the GP is capable of). Despite the barriers, GPs agreed that AK care is largely a responsibility of primary care, except for the treatment of persistent and therapy-resistant lesions. Transition from hospital to primary care for patients with AK was supported by all GPs and was not expected to increase their workload enormously. Some GPs argued that in the future, AK care could partly be delegated to physician assistants and nurse practitioners. One GP opted for accreditation of GPs with extended roles in dermatology.

## Discussion

This quantitative study based on semi-structured in-depth interviews shows various AK management approaches amongst Dutch GPs with suboptimal guideline compliance due to diverse underlying barriers. They reported a need for more (continuing) education, especially on 5-FU. The main challenges in AK management as perceived by GPs were dealing with diagnostic uncertainty, treating extensive lesions, managing treatment-related skin reactions, and reconciling patient misconceptions. Despite the barriers, GPs agreed that AK care is largely a responsibility of primary care and supported the transition from hospital to primary care.

### Comparison with national guideline and previous studies

#### Management of AK

On a number of points, the current practice of the GPs interviewed in this study differs from the DCGP guideline on ‘suspicious skin abnormalities’. First, it is advised to choose between cryotherapy or 5-FU therapy in consultation with the patient if treatment is desired or necessary [14]. Instead, most GPs apply cryotherapy without discussing other treatment options, including the option of no treatment. A similar finding was obtained in a qualitative study among 22 Dutch GPs, who were interviewed prior to issuing of the DCGP guideline in 2017 [13]. The fact that cryotherapy is simple, non-invasive and easily applicable might explain the preference of GPs for this treatment over 5-FU. In general, financial incentives may influence treatment selection. However, it is unlikely that this affects AK management in primary care in the Netherlands, because there is no difference in remuneration for Dutch GPs between cryotherapy and 5-FU. Second, it is advised to evaluate the treatment effect after 3 months [14]. However, most GPs do not routinely schedule a follow-up visit after cryotherapy, but advise the patient to book a follow-up appointment if healing failed to occur. However, regular post-treatment follow-up might provide GPs of essential feedback to further expand their treatment experience. Third, referral to a dermatologist is only advised for patients in whom the treatment response is incomplete or for whom none of the first-line treatment modalities are appropriate [14]. Although a recent survey among 100 GPs showed that 1 in 3 GPs have started to treat more patients with AK since the issuing of the DCGP guideline [21], the current study indicates that in case of optimal guideline adherence, the number of referrals to a dermatologist is expected to further decrease.

### Needs and challenges

The GPs interviewed in this study report a need for more dermatology (continuing) education, which was also reported in previous studies [13, 22]. It was even suggested that dermatology be made a compulsory course to ensure that dermatology education at all medical schools in the Netherlands is equal and of sufficient quality [23]. Currently, national agreements are lacking and each GP training institute determines separately how much attention is paid to dermatology education. It is a priority to increase dermatological knowledge among GPs and GP residents [24].

The need for more dermatology (continuing) education is closely related to dealing with diagnostic uncertainty, one of the four main challenges that GPs in this study perceive in AK management. A recent observational study showed that GPs who followed a dermato-oncological training programme had better diagnostic skills and quality of referrals than untrained GPs, leading to fewer potentially unnecessary referrals [25]. This may also lead to a more efficient utilization of hospital care and lower healthcare costs.

The second and third main challenges relate to treating extensive lesions and managing treatment-related skin reactions. In patients with extensive lesions, most topical treatment options require prolonged use and cause a local inflammatory response that limits tolerability and adherence, which may result in underutilization of topical treatments [26]. GPs are open to the use of other treatment modalities, such as daylight photodynamic therapy, which might assist in overcoming the mentioned challenges. This treatment modality is included in the guideline on ‘actinic keratosis’ from the Dutch Society for Dermatology and Venereology as one of the field-directed treatment modalities to consider based on compliance, ease of application, patient preferences and history [27]. Daylight photodynamic therapy has been available in primary care since 2021, although limitedly applied and not recommended in the 2017 DCGP guideline.

A fourth main challenge relates to reconciling patient misconceptions, for example regarding local skin reactions related to 5-FU therapy or a request for direct referral without knowing what the GP is capable of. Although the DCGP guideline clearly describes the importance of providing sufficient patient information [14], this is not always easy in daily practice [28, 29]. Previously evaluated initiatives include transmural patient information leaflets, decision aids, and educational videos, which may warrant further investigation [25, 29, 30].

Despite the barriers, the GPs interviewed in this study agree that AK care is largely a responsibility of primary care. This finding is consistent with the results of a survey in which 268 Dutch GPs were asked about their views and opinions on the role of GPs in skin cancer care.^22^ 75% of respondents reported AK treatment to be a task for GPs. Experience with 5-FU therapy was very limited (16%), as was the case with GPs in the current study. However, 56% of respondents were willing to treat patients themselves if they had more knowledge of it.

### Strengths and limitations

The major strength of this study is that it provides profound knowledge of AK care provided by GPs, specifically focusing on the needs and challenges in AK management after the implementation of the DCGP guideline in 2017. Although this study was conducted within the context of the Dutch healthcare system, its results are transferable to other countries in which GPs act as ‘gatekeepers’. Two limitations should be considered in interpreting the findings of this study. First, participants were selected from a convenience sample of GPs subscribed to an online learning platform for dermoscopy in primary care and this sample was nonrandomly drawn. One may argue that it is likely that the study population had a higher-than-normal interest in dermatology and that this may hamper the generalizability of the results. Also, one could suppose that average GPs may have more needs and challenges. However, the purpose of qualitative research is to provide in-depth explanations and meanings, rather than generalizing findings. [31] A previous qualitative study on AK management among Dutch GPs and dermatologists used a similar approach [13], therefore allowing comparison of results. Second, transcripts were coded and analysed by only one researcher due to limited resources. This may have led to some interpretation bias, but the researcher was assisted by experienced staff members (ZC and professor Isabelle Huys, KU Leuven).

## Conclusion

This qualitative study shows that AK management varies considerably among Dutch GPs and compliance with the national guideline is suboptimal due to a variety of reasons and barriers. The main challenges in AK management as perceived by GPs were dealing with diagnostic uncertainty, treating extensive lesions, managing treatment-related skin reactions, and reconciling patient misconceptions. More standardised and uniform AK management requires more (continuing) education, especially on 5-FU therapy. Despite the barriers, GPs agreed that AK care is largely a responsibility of primary care and supported further transition from hospital to primary care, in line with current guideline recommendations.

### Electronic supplementary material

Below is the link to the electronic supplementary material.

Supplementary Material 1

## Data Availability

The dataset used and/or analysed during the current study is available from the corresponding author on reasonable request.

## Abbreviations

5-FU: 5-fluorouracil
AK: Actinic keratosis
DCGP: Dutch College of General Practitioners
GP: General practitioner
