# Supplementary material for: Needs and challenges among general practitioners in the management of actinic keratosis: a qualitative study
Source: BMC Prim Care. 2023 Dec 2;24:260. doi: 10.1186/s12875-023-02202-6 (PMC10693056; doi:10.1186/s12875-023-02202-6)
Supplement: Supplementary file 1 — Supplementary Material 1 [file 12875_2023_2202_MOESM1_ESM.docx]

**Supplementary Material.** Interview guide used to explore the needs and challenges among Dutch general practitioners in the management of actinic keratosis.

*I. General information*

- On average, how many patients consult you with (suspected) actinic keratosis (AK) each week/month?
- How do you rate your knowledge and skills for diagnosis and treatment of AK?
- Do you think available (continuing) education is sufficient to improve your knowledge on skin cancer and AK?
  - If not, what further training would you need?
- Should patients with AK be managed in primary or hospital care? If primary care:
  - What is needed to improve the management of AK in primary care?
  - How would you organize the management of AK in primary care?
- Are you familiar with the national guideline on ‘suspicious skin abnormalities’ from the Dutch College of General Practitioners (DCGP), and to what extend do you adhere to this guideline?

*II. Diagnosis*

- How do you diagnose AK?
- Do you diagnose AK yourself and if so, how certain are you of this diagnosis? For example, can you properly distinguish AK from non-melanoma skin cancer?
- How well do you manage to grade the severity of AK lesions?
- What are reasons for you to perform a biopsy in patients with (suspected) AK?
- What percentage of patients with AK do you refer to a dermatologist, and what is your main reason for referral?
- What challenges do you experience in diagnosing AK, and what could be ways to overcome these challenges?

*III. Treatment*

- If you choose to treat a patient with AK yourself, what treatment do you prefer, and why?
- Is your choice of treatment based on certain disease characteristics (e.g., solitary vs. multiple lesions, severity, localization)?
- Do you consider the recommended treatment options in the DCGP guideline (i.e., cryotherapy, 5-fluorouracil) as sufficient?
  - If not, what are the therapeutic needs for you and your patients, and why?
- How do you involve the patient in treatment decisions?
  - What role does patient preference play in treatment decisions?
- What challenges do you experience in treating AK, and what could be ways to overcome these challenges?
- Are you familiar with daylight photodynamic therapy?
  - Are you open to the use of daylight photodynamic therapy in your practice, and why (not)?
  - Do you think daylight photodynamic therapy can be of added value to the management of AK in primary care?

*IV. Follow-up*

- How do you follow up with patients with AK?
  - Do you routinely schedule a check-up appointment after 3 months?
- What do you do if the intial treatment is ineffective?
